# Vector Competence of Northern European *Culex pipiens* Biotype *pipiens* and *Culex torrentium* to West Nile Virus and Sindbis Virus

**DOI:** 10.3390/v15030592

**Published:** 2023-02-21

**Authors:** Stephanie Jansen, Anna Heitmann, Ruut Uusitalo, Essi M. Korhonen, Renke Lühken, Konstantin Kliemke, Unchana Lange, Michelle Helms, Lauri Kirjalainen, Roope Nykänen, Hilppa Gregow, Pentti Pirinen, Giada Rossini, Olli Vapalahti, Jonas Schmidt-Chanasit, Eili Huhtamo

**Affiliations:** 1 Bernhard Nocht Institute for Tropical Medicine, 20359 Hamburg, Germany; 2 Faculty of Mathematics, Informatics and Natural Sciences, University of Hamburg, 20146 Hamburg, Germany; 3 Department of Virology, Medicum, University of Helsinki, 00100 Helsinki, Finland; 4 Department of Veterinary Biosciences, University of Helsinki, 00100 Helsinki, Finland; 5 Department of Geosciences and Geography, University of Helsinki, 00100 Helsinki, Finland; 6 Finnish Meteorological Institute, 00101 Helsinki, Finland; 7 Unit of Microbiology, Department of Experimental, Diagnostic and Specialty Medicine, University of Bologna, 40138 Bologna, Italy; 8 Virology and Immunology, Diagnostic Center, Helsinki University Hospital (HUSLAB), 00290 Helsinki, Finland

**Keywords:** vector-competence, *Culex pipiens*, *Culex torrentium*, West Nile virus, Sindbis virus, Finland

## Abstract

The West Nile Virus (WNV) and Sindbis virus (SINV) are avian-hosted mosquito-borne zoonotic viruses that co-circulate in some geographical areas and share vector species such as *Culex pipiens* and *Culex torrentium*. These are widespread in Europe, including northern parts and Finland, where SINV is endemic, but WNV is currently not. As WNV is spreading northwards in Europe, we wanted to assess the experimental vector competence of Finnish *Culex pipiens* and *Culex torrentium* mosquitoes to WNV and SINV in different temperature profiles. Both mosquito species were found susceptible to both viruses and got infected via infectious blood meal at a mean temperature of 18 °C. WNV-positive saliva was detected at a mean temperature of 24 °C, whereas SINV-positive saliva was detected already at a mean temperature of 18 °C. *Cx*. *torrentium* was found to be a more efficient vector for WNV and SINV over *Cx*. *pipiens*. Overall, the results were in line with the previous studies performed with more southern vector populations. The current climate does not seem optimal for WNV circulation in Finland, but temporary summertime transmission could occur in the future if all other essential factors are in place. More field data would be needed for monitoring and understanding the northward spreading of WNV in Europe.

## 1. Introduction

The ongoing climate and environmental changes are affecting the distribution and abundance of mosquitoes and, subsequently, the occurrence of diseases caused by mosquito-borne viruses (MBV) globally [1]. Finland is endemic to the mosquito-borne Sindbis virus (SINV), which is an alphavirus endemic in Eurasia, Africa and Australia. The human disease febrile rash-arthritis is reported with especially high incidence in Finland [2]. SINV has caused several outbreaks since its introduction to Finland in the 1970s, the largest in 1995, with 1311 diagnosed cases [3]. The recent extremely warm summers in Finland have coincided with the increased epidemic activity of SINV in 2018 [4] and in 2021, when an outbreak with over 500 human cases [5] was reported. This raised the question of whether a warming climate with frequent and extreme heat waves at northern latitudes would also enable new mosquito-borne viruses, such as West Nile virus (WNV), to be introduced to Finland. West Nile virus (WNV), a member of the genus Flavivirus in the family *Flaviviridae*, is clinically the most significant MBV in Europe. WNV is globally widespread and causes febrile diseases of varying severity, including severe central nervous system infections in humans and horses [6]. The largest WNV outbreak in Europe occurred in 2018, associated with unusually warm weather, with 1993 reported cases, most of them occurring in August [7]. WNV is currently spreading northwards in Europe, with recent reports from Germany [8,9] and the Netherlands [10].

SINV and WNV both have a bird-*Culex* mosquito ecological cycle and are co-circulating in areas of South Africa [11] and Israel [12], and likely also in Europe [13], although currently, data is lacking on the exact regions where this occurs. The global phylogeny of SINV suggests a likely introduction to northern Europe via migratory birds from Africa [14]. The introduction of also WNV via migratory birds to northern Europe seems possible, although available serological data has suggested low bird exposure [15]. Several mosquito vector species have been associated with WNV and SINV [13,16]; however, they are known to be vectored by the same Culex species, namely, the *Culex pipiens* biotype *pipiens* and *Culex torrentium* that also vector other viruses such as Usutu virus. These vector species are widespread in Europe all the way from the south to the northern parts [17], including Finland [18] and Sweden [19].

For assessing the potential risk for local transmission of WNV in Finland, we tested the vector competence of Finnish *Culex pipiens* biotype *pipiens* and *Culex torrentium* mosquitoes for WNV and SINV. In addition, we studied the recent and future climate estimations for Finland together with the experimental vector competence data.

## 2. Materials and Methods

### 2.1. Collection and Rearing of Mosquitoes

Mosquito egg rafts were collected from artificial water containers in 2019 and 2021 in Helsinki, Finland (60°11′ N, 24°56′ E). The vector competence experimental setup and methodologies were based on previous studies on German mosquitoes [20,21]. In brief, mosquito egg rafts were individually reared and genetically identified [20]. Before the experimentation, a subset of mosquitoes was screened for pan-flavi, pan-alpha and pan- orthobunyavirus RNA with previously published protocols [22,23,24].

### 2.2. Infection of Mosquitoes

Mosquitoes were fed with artificial bloodmeal ((50% human blood (expired blood preservation), 30% fructose (8% solution), 10% filtrated bovine serum (FBS) and 10% virus solution) containing WNV (clade 1a, strain TOS-09, Genbank HM991273/HM641225, passage 5 from Vero cells) or SINV (lineage I, BNI-10865, Genbank MF 589985.1, passage 6) at a final titer 10^7^ plaque-forming units per milliliter (PFU/mL).

### 2.3. Vector Competence Assay

To determine the vector competence, in terms of the general ability of mosquitoes to transmit a virus to saliva, we performed mosquito infection experiments for Finnish Culex mosquitoes and tested the saliva samples for the presence of the viable virus. In brief, 7–10-day-old female mosquitoes were fed with an artificial bloodmeal containing WNV or SINV according to a previously described protocol [20]. Fully engorged females were incubated at a relative humidity of 70% with a 12 h:12 h light and dark rhythm under different temperature profiles. Mosquitoes were incubated at evenly oscillating temperature profiles with variations of ±5 °C within 24 h to mimic day-night temperature variations with mean temperatures of 18, 21, 24 and 27 °C, as previously described for both viruses [16,21]. Due to the limited number of mosquitoes, SINV was only tested at two temperature profiles, 18 ± 5 °C and 24 ± 5 °C (mean 18 and 24 °C), that were previously shown to be sufficient for German Culex species to transmit SINV [16]. Salivation assay was performed at 5 days post-infection (dpi) for SINV and 14 dpi for WNV as previously described [16,21]. In brief, harvested saliva was tested for the presence of infectious virus particles using Vero cells. Cells seeded in a 96-well plate were incubated with saliva samples, and the cytopathic effect (CPE) was monitored for 5 days in SINV experiments and for 7 days in experiments with WNV. For confirming the virus replication, the supernatants of CPE-positive samples were additionally tested for WNV RNA using Real Star WNV RT-PCR Kit (Altona diagnostics, Hamburg, Germany) and for SINV RNA using a qRT-PCR method [21,25] including VetMAX™ Xeno™ internal positive control (Applied Biosystems, Waltham, MA, USA) according to the manufacturer’s protocol. The transmission efficiency (TE, the number of SINV or WNV-positive saliva per the number of fed females) was then calculated. The transmission rate (TR, the number of positive saliva per the number of viral RNA-positive bodies) was then calculated. For assessing the infection rate and viral copy numbers in the infected mosquitoes, body specimens were homogenized, and RNA was extracted and tested for viral RNA, as described above. The infection rate (IR, the number of viral RNA-positive bodies per all fed mosquitoes) and the mean viral RNA amount per body was calculated.

### 2.4. Air Temperature and Relative Humidity Data

Recent air temperature and relative humidity (RH) data were obtained from the Finnish Meteorological Institute (FMI) [26] and examined for conditions matching the experiments. A number of days (of at least 14 consecutive days) were studied for daily mean air temperatures of 18 °C and 21 °C and for mean RH of ≥70% during the summer season, June–August, in Finland in 2021 at a 1 km resolution.

Future climatic data were derived from CHELSA V2 using IPSL-CM6A-LR global climate model (GCM) with the shared socioeconomic pathway (SSP) scenarios and the representative concentration pathways (RCPs) of SSP3-RCP7 and SSP5-RCP8.5 [27]. Data were extracted for Finland and included projected mean monthly temperatures in July, the warmest month in the country, for 2041–2070 and 2071–2100. Maps were created using ESRI ArcGIS (version 10.3.1; ESRI, Redlands, CA, USA).

## 3. Results

**Figure 1:**
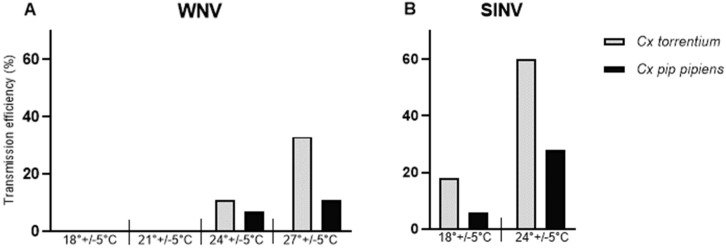
Transmission efficiency (TE) of *Culex torrentium* and *Culex pipiens* biotype *pipiens* at 70% humidity and different temperature profiles for (**A**) West Nile virus (WNV, 14 dpi) at an MOI of 10^7^ PFU/mL) and (**B**) Sindbis virus (SINV, 5 days post-infection (dpi).

Reared mosquitoes included species *Culex pipiens* biotype *pipiens* and *Culex torrentium,* and the subset tested in pan-arbovirus screening RT-PCRs was found negative. The survival rate of the mosquitoes was mostly 100% across the experiment, independent of species and virus used. In comparison to other tested temperature profiles, a slight decrease in survival of *Culex torrentium* was observed at 27 °C after 14 days. Both mosquito species were successfully infected with SINV at both tested temperatures (Table 1). All CPE were confirmed by the respective qRT-PCR to be virus positive. In *Culex pipiens,* the SINV IRs were 48% at 24 °C and 23% at 18 °C. In *Culex torrentium,* SINV IRs were higher, reaching 87% at 24 °C and even 97% at 18 °C. Transmission of SINV was observed at both temperatures, with higher TRs and TEs at 24 °C than at 18 °C. *Culex torrentium* had higher transmission efficiencies for SINV (TE of 60% at 24 °C) compared to *Culex pipiens* biotype *pipiens* (TE of 28% at 24 °C; Table 1, Figure 1).

WNV infection was detectable for both *Culex* species at all four temperature profiles (18, 21, 24 and 27 °C). Mean viral RNA body titers were highest at the highest used temperature profile (27 °C), reaching 8 log10 genomic RNA copies for *Culex torrentium* and 7.85 log10 for *Culex pipiens* biotype *pipiens.* Transmission was only observed at the two highest temperature profiles tested; 27 °C and 24 °C (Table 1, Figure 1). *Culex torrentium* reached a TE of 33% at the highest temperature compared to 17% for *Culex pipiens* biotype *pipiens* (Table 1). Notably, also the infection rate of *Culex torrentium* was higher (97%) than *Culex pipiens* biotype *pipiens* (63%) at the lowest tested temperature (18 °C).

**Figure 2:**
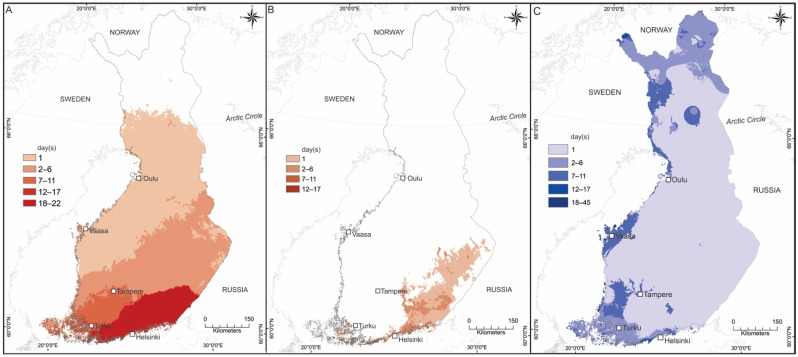
Mean air temperatures of (**A**) 18 °C, (**B**) 21 °C and (**C**) mean RH ≥ 70% in 2021 corresponding to experiment duration of 14 consecutive days (day(s) = 1). Number of additional days (after 14 consecutive days, day(s) ≥ 2) are indicated with a color gradient.

Air temperature and RH data for Finland in 2021 were compared with the vector competence experiment. The oscillating temperature profiles with ±5 °C within 24 h approximately corresponded to the observed summer months’ mean daily temperature fluctuation of 10.4 °C [26]. However, the observed air temperature data for Finland in 2021 did not indicate periods corresponding to those found experimentally suitable for WNV transmission in this study: at least 14 consecutive days at daily mean air temperatures of 24 °C or 27 °C. However, corresponding periods with daily mean air temperatures of 18 °C and 21 °C were observed (Figure 2A,B) in which mosquitoes were experimentally infected with WNV. Although only the mean air temperatures were considered and compared here, it was noted that the air temperature data of 2021 showed consecutive daily maximum air temperatures of 22–32 °C in parts of southern Finland up to 62° N latitude for a duration of over 4 weeks (data not shown). Based on the future climate data with both SSP3-RCP7 and SSP5-RCP8.5 scenarios, the estimated monthly mean temperature of >24 °C in July was expected mainly in regions located in southern and eastern Finland by the end of the 21st century (Supplementary Figure S1). In the summer of 2021, the mean RH was as it was in the experiment, 70% or beyond, for at least 14 consecutive days in nearly the whole country (Figure 2C).

## 4. Discussion

The experimental data on the vector competence of Culex mosquitoes for WNV from different climatic zones is scarce and has been lacking from the northernmost parts of Europe. We assessed this by studying the vector competence of Finnish *Culex pipiens* biotype *pipiens* and *Culex torrentium*. We used WNV and SINV strains that were previously tested using German mosquitoes in the same laboratory [16,20]. We focused on the influence of the temperature and therefore used rigid humidity conditions of 70% as well as incubation periods that have been shown to lie within the extrinsic incubation period of the used virus [16,20]. Our results demonstrate that these mosquito species collected from Helsinki, Finland, located further north than populations studied before in Europe [20,28], are competent vectors for WNV.

We included SINV in the study as a control virus and to test the overall arbovirus vectoring ability of the collected mosquitoes. The results for SINV were in line with previous studies [16], including vector competence experiments performed using mosquitoes from a neighboring country, Sweden [29]. In this study, SINV was transmitted at 18 °C, whereas WNV required 24 °C to be detectable in saliva. Although only a small number of specimens were available for this study, differences in the transmission efficiencies were noted between the two Culex species. *Culex torrentium* from Finland appeared to be a more competent vector for WNV and SINV compared to *Culex pipiens* biotype *pipiens*. This is in line with previous studies that demonstrated *Culex torrentium* from Germany to be more efficient as a vector for WNV and SINV [16,20] and *Culex torrentium* from Sweden for SINV [29] in comparison to *Culex pipiens*. Additionally, *Culex torrentium* mosquitoes were more efficiently infected with WNV and SINV at low temperatures (18 °C) than *Culex pipiens* biotype *pipiens*. Although in this study, no transmission of WNV (Italian lineage 1 strain) was detected at 18 °C, this has been documented in German *Culex pipiens* biotype *pipiens* using German WNV lineage 2 [30]. Currently, the minimum temperature thresholds for WNV and its required minimum duration are not known for European *Culex pipiens* biotype *pipiens* and *Culex torrentium* and may also be affected by virus strain properties.

The more efficient vectoring ability of *Culex torrentium* is interesting as it also appears to be the dominant Culex species and geographically more widespread in Finland and Sweden than more southern *Culex pipiens* biotype *pipiens* [18,19]. The abundance of these two vectors in urban Helsinki indicates the potential for urban mosquito-borne arbovirus transmission, although Helsinki is not included in the predicted risk areas for SINV [31]. In Finland, the risk areas for endemic SINV include habitats that support resident grouse populations that are not present in southern coastal areas.

In Finland and elsewhere in northern Europe, the warmest summer months represent a time window where possible conditions for WNV transmission could take place. In Helsinki, in locations where the mosquitoes for this study were collected, Culex egg rafts and larvae are found in artificial water containers generally from late June towards the end of the mosquito season, the main flying season probably being in June–August when also the warmest summer weather is usually experienced. The observations from the record warm summer of 2021 in Finland showed that although the mean RH data suggested 70% or above occurred widely in the country for the duration of at least 14 consecutive days, with temperature and duration matching strictly the experimental settings where WNV was experimentally transmitted by Finnish Culex mosquitoes had not occurred. But matching conditions did occur in which the local Culex mosquitoes could have been infected with WNV, e.g., if a viremic bird had been available as the source of a blood meal. The regions in which daily mean air temperatures of 21 °C at least 14 consecutive days were observed included narrow areas on the southern coast located close to densely populated regions such as the capital region of Helsinki with many entry points such as airports and harbors. It was noted that although daily maximum temperatures in Finland were very high for several weeks in a row in July–August 2021, the mean temperatures remained modest due to large daily temperature fluctuations. This raised questions about the possible underestimates of the climatic suitabilities based on experimental mean temperatures and experiment durations.

The large temperature fluctuations occurring in nature probably have an effect on virus replication in a mosquito and virus dissemination. The extremes of these conditions occur during virus overwintering, which can be considered a key factor for WNV spreading northwards. WNV overwintering has been demonstrated in European *Culex pipiens* [32,33]. WNV tolerance for suboptimal temperatures has also been demonstrated in simulated overwintering followed by returning replication and transmissibility in New York *Culex pipiens* [34] and in Chinese *Culex pipiens pallens* [35].

The current climate in Finland is not likely to support an efficient WNV transmission cycle. Although the future climate estimates suggest temperatures supporting temporal summer-time WNV transmission in parts of the country by the end of the 21st century, Finland is not likely to be among the first areas at risk. The record warm summer in 2021 is one indication of the effects of climate change at northern latitudes. Longer and more extreme heatwaves are expected to be more frequent in the future, and elsewhere, WNV transmission has been found to disperse into new areas, specifically during summers with above-average temperatures [36].

The geographical distribution of WNV in Europe is not uniform, highlighting the complexity of the factors affecting WNV presence or absence. Suitable climatic conditions and the presence of competent vectors are not the only requirements. Occurrence of introduction, timing and availability of competent vectors and susceptible vertebrate hosts in supporting climatic conditions that enable the enzootic transmission cycle are needed. We consider the introduction of WNV to northern Europe and Finland possible via natural bird migration from endemic areas in Europe or Africa. This does not seem very likely, taking into account that, in Finland, the main bird spring migration occurs in March to May when it is likely to be too cold for transmission and very few mosquitoes are observed flying. Nevertheless, it is considered that, for example, SINV has spread to northern Europe and Finland via migratory birds under these conditions [14]. In addition to this natural introduction, human activity could also import infected avian hosts or vectors. Even in such a case, the starting of a local transmission chain would depend on multiple factors that would need to be favorably aligned.

Taken together, competent vectors for WNV are present in Finland and likely wider in northern parts of Europe. Further northwards spread of WNV seems possible and requires monitoring also in temperate climates. As only a limited amount of experimental conditions can be tested, and those may not well reflect real-life situations, further field data would be needed to elucidate the drivers and limiting factors of WNV for assessing future risks in Europe.

## Figures and Tables

**Table 1:** Infection rates (IR), viral RNA copy number/body (Mean (95% confidence interval) log10 RNA copies/specimen), transmission rates (TR) and transmission efficiency (TE) of *Culex torrentium* and *Culex pipiens* biotype *pipiens* at 70% humidity and different temperatures for arbovirus infection with Sindbis virus (SINV, 5 days post-infection (dpi) or West Nile virus (WNV, 14 dpi) at an MOI of 10^7^ PFU/mL). *n* = number of investigated specimens.

Virus	Mosquito Species	Temperature	Dpi	*n*	IR	Viral RNA Copy Number/Body	TR	TE
WNV	*Culex torrentium*	27 ± 5 °C	14	24	92% (22/24)	8.00 (7.25–8.75)	36% (8/22)	33% (8/24)
		24 ± 5 °C	14	9	100% (9/9)	7.35 (6.51–8.20)	11% (1/9)	11% (1/9)
		21 ± 5 °C	14	30	83% (25/30)	6.93 (6.12–7.73)	0% (0/25)	0% (0/30)
		18 ± 5 °C	14	32	97% (31/32)	6.01 (5.37–6.65)	0% (0/31)	0% (0/32)
	*Culex pipiens pipiens*	27 ± 5 °C	14	35	43% (15/35)	7.85 (6.66–9.04)	40% (6/15)	17% (6/35)
		24 ± 5 °C	14	30	83% (25/30)	7.28 (6.46–8.11)	8% (2/25)	7% (2/30)
		21 ± 5 °C	14	30	73% (22/30)	5.99 (5.26–6.71)	0% (0/22)	0% (0/30)
		18 ± 5 °C	14	30	63% (19/30)	5.72 (4.98–6.45)	0% (0/19)	0% (0/30)
SINV	*Culex torrentium*	24 ± 5 °C	5	30	87% (26/30)	7.81 (6.98–8.65)	69% (18/26)	60% (18/30)
		18 ± 5 °C	5	34	97% (33/34)	7.73 (7.19–8.62)	18% (6/33)	18% (6/34)
	*Culex pipiens pipiens*	24 ± 5 °C	5	29	48% (14/29)	5.16 (3.90–6.43)	57% (8/14)	28% (8/29)
		18 ± 5 °C	5	31	23% (7/31)	4.33 (3.52–5.14)	29% (2/7)	6% (2/31)

## Data Availability

Not applicable.

## Supplementary Materials

The following supporting information can be downloaded at: https://www.mdpi.com/article/10.3390/v15030592/s1.
