# Supplementary figures and images for: Vector Competence of Northern European Culex pipiens Biotype pipiens and Culex torrentium to West Nile Virus and Sindbis Virus
Source: Viruses. 2023 Feb 21;15(3):592. doi: 10.3390/v15030592 (PMC10056470; doi:10.3390/v15030592)

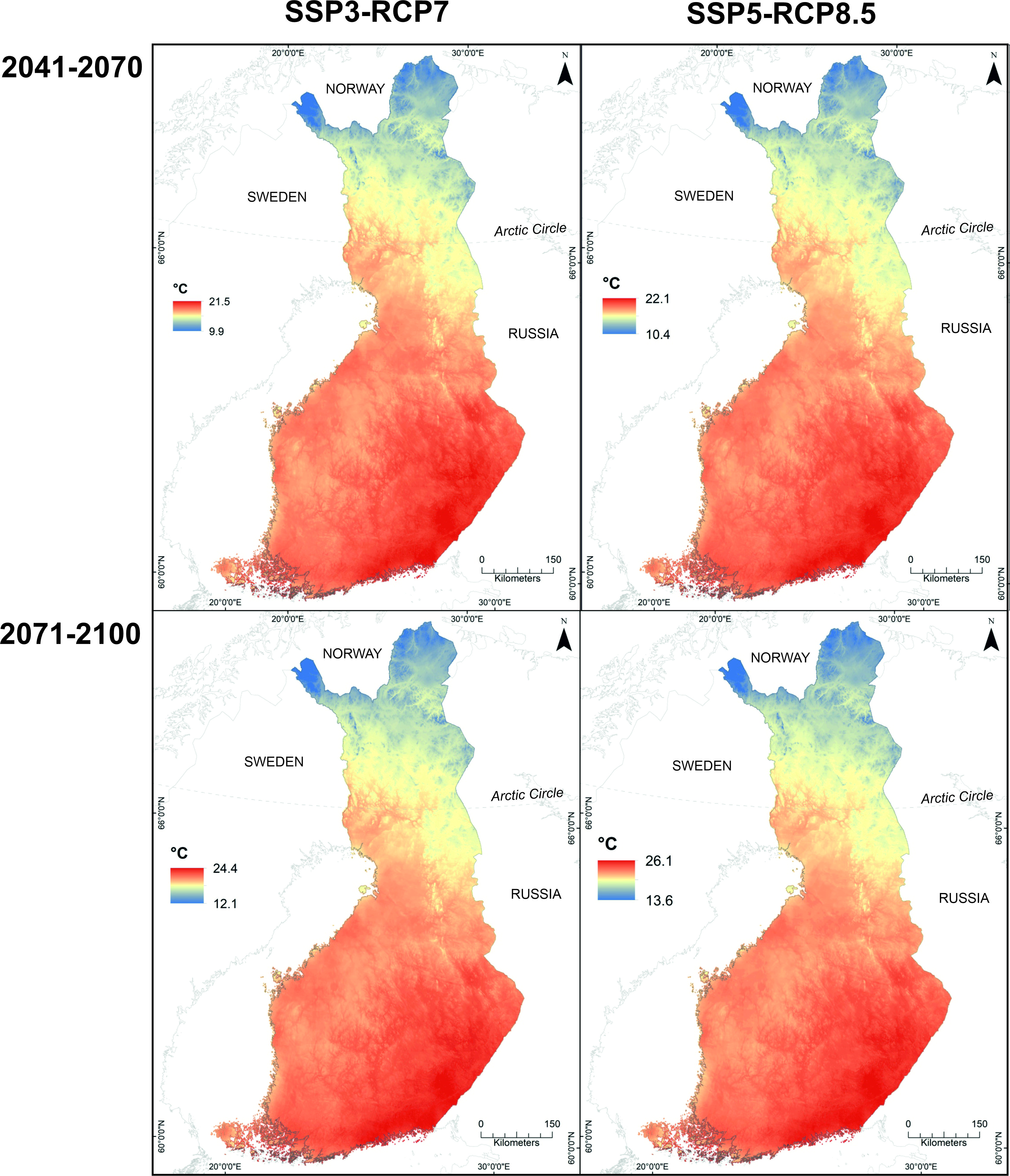

Supplement: Supplementary file 1 [file viruses-15-00592-s001.zip › viruses-2028511-supplementary.jpg]
